# Mouse brain MR super-resolution using a deep learning network trained with optical imaging data

**DOI:** 10.3389/fradi.2023.1155866

**Published:** 2023-05-15

**Authors:** Zifei Liang, Jiangyang Zhang

**Affiliations:** Department of Radiology, Center for Biomedical Imaging, New York University, New York, NY, United States

**Keywords:** MRI, super-resolution (SR), deep learning, transfer learning, multi-modality image

## Abstract

**Introduction:**

The resolution of magnetic resonance imaging is often limited at the millimeter level due to its inherent signal-to-noise disadvantage compared to other imaging modalities. Super-resolution (SR) of MRI data aims to enhance its resolution and diagnostic value. While deep learning-based SR has shown potential, its applications in MRI remain limited, especially for preclinical MRI, where large high-resolution MRI datasets for training are often lacking.

**Methods:**

In this study, we first used high-resolution mouse brain auto-fluorescence (AF) data acquired using serial two-photon tomography (STPT) to examine the performance of deep learning-based SR for mouse brain images.

**Results:**

We found that the best SR performance was obtained when the resolutions of training and target data were matched. We then applied the network trained using AF data to MRI data of the mouse brain, and found that the performance of the SR network depended on the tissue contrast presented in the MRI data. Using transfer learning and a limited set of high-resolution mouse brain MRI data, we were able to fine-tune the initial network trained using AF to enhance the resolution of MRI data.

**Discussion:**

Our results suggest that deep learning SR networks trained using high-resolution data of a different modality can be applied to MRI data after transfer learning.

## 1. Introduction

Magnetic resonance imaging (MRI) is a non-invasive imaging technique with many applications in neuroscience research, both in humans and animals, due to its rich soft tissue contrasts. For example, T1- and T2-weighted MRI are commonly used to examine brain structures, and diffusion MRI (dMRI) is sensitive to tissue microstructure and useful for detecting acute stroke (1). Compared to other imaging techniques, such as optical imaging, MRI is inherently a low signal-to-noise (SNR) technique, and as a result, its spatial resolution is limited at the millimeter (mm) or sub-mm level. Advances in high-field MRI and high-performance gradient systems have greatly improved the spatial resolution of MRI but these approaches are facing increasing challenges associated with the high complexity and cost of such systems (2–5).

A promising approach to improving resolution is using super-resolution (SR) to transform a low-resolution image into a high-resolution image. The conventional machine-learning-based image SR takes multiple images acquired with sub-voxel spatial shifts and produces images with higher spatial resolution (6–12). For example, in order to improve the resolution along the slice direction, multiple images can be acquired, each shifted by a known sub-voxel distance along the slice direction, and images with enhanced through-plane resolution can be reconstructed (13). To improve in-plane resolution, acquiring multiple images with rotated scans has also been reported (14, 15). Also, some work incorporated extra information from inter-scan subject motions or image distortions to achieve SR (6, 16). These MRI SR methods have many applications, such as generating finer maps of metabolic activities of the brain (9), improving brain structure segmentation (6), or visualizing small white matter tract (17). However, the need to acquire multiple images increases the total imaging time and makes the technique susceptible to motions (11). An alternative SR approach is based on sparse representation (18–20), but is sensitive to noise and is computationally expansive.

When conventional and sparse representation-based SR are not applicable, deep learning-based SR has been proposed as an alternative (21). Leveraging large amounts of training data, many reports have demonstrated the success of this approach using generic images. Early work used the Feed-Forward neural networks, including the convolutional neural network (CNN) [e.g., the LeNet5 proposed in 1998 by LeCun et al. (22)], to achieve image SR (21), but used moderate numbers of layers and neurons, which limited its performance. Later, the ResNet further improved the performance by using “skip connections” to avoid the problem of vanishing gradients in very deep (>100) networks (23). Recent development based on generative adversarial network (GAN) provided additional improvements (24). Deep learning based SR of MRI data has been demonstrated recently using CNN, ResNet, and GAN-based networks (25–29), which showed success in improving results from low-field MRI systems (30), dMRI studies (31, 32), as well as coronary MR angiography (33).

Although deep-learning-based SR has made a lot of progress in recent years, especially on generic images, it is still not fully understood how image contrast and resolution affect the performance of SR. Some work already analyzed the effects of resolution on deep learning performance (e.g., 34), on diagnosis or image labeling tasks. There have been several reports that took advantage of multiple MRI contrasts for a variety of tasks including image segmentation, diagnosis, and lesion detection (35–39), using the multi-contrast information to train a neural network. Many SR methods take multi-contrast MRI data to train a neural network or use them as a prior/constraint for SR (40–43). This approach allows the network to learn the different characteristics of each contrast and how they relate to the task at hand, such as (40), which used high-resolution T1-weighted MRI data to enhance the resolution of low resolution T2-weighted MRI data.

Large collections of high-resolution MRI data of the human brain, such as the Human Connectome Project (HCP) (44), which contain hundreds of MRI data acquired with identical resolutions and contrasts, made it possible to train deep learning network to perform SR (45, 46). In contrast, studies involving the mouse brain often have a much smaller sample size, and pooling data from multiple studies is not a viable option due to variations in imaging resolution and contrasts caused by differences in study designs and MRI systems. While similar MRI resources for the mouse brain still do not exist, more than 1,700 three-dimensional (3D) mouse brain auto-fluorescence (AF) images acquired using optical imaging techniques [e.g., serial two-photon tomography (STPT)] are available from the Allen mouse brain connectivity atlas (AMBCA) (47). In this study, using the large collection of 3D AF data, we examined how much improvement deep learning based SR can provide for mouse brain data and whether we can leverage the AMBCA mouse brain AF data to enhance the resolution of mouse brain MRI data. We also examined the effects of contrasts and resolution of the training data on deep learning-based SR. Specifically, we investigated the difference between training using low and high-resolution AF data, applying SR networks trained using AF data to MRI data, and using transfer learning to adapt networks trained using AF data to different MRI contrasts.

## 2. Method and material

### 2.1. Auto-fluorescence (AF) data of the mouse brain

**Figure 1:**
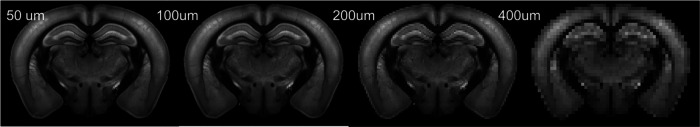
Representative down-sampled AF images of a mouse brain. This axial section was selected from a 3D AF volume.

High-resolution 3D auto-fluorescence AF data (*n* = 100), with a spatial resolution of 25 µm × 25 µm × 25 µm were downloaded from AMBCA (http://help.brain-map.org/display/mousebrain/API). These data were used as the training and testing data in the following sections. 3D AF data were down-sampled by averaging the corresponding patch voxels to various resolutions (Figure 1).

### 2.2. Animals MRI

We acquired multi-contrast mouse brain MRI data to examine the performance of SR networks trained using 3D AF data. All animal experiments have been approved by the Institute Animal Care and Use Committee at New York University. Inbred C57BL/6 mice (Jackson Laboratories, 4 months old, female, *n* = 10) were fixed by trans-cardiac perfusion of 4% paraformaldehyde in phosphate buffered saline (PBS) for *ex vivo* MRI. After fixation, mouse heads were removed and immersed in 4% PFA in PBS for 24 h at 4°C before being transferred to PBS. Before *ex vivo* MRI, specimens were placed into 10 ml syringes, which were filled with Fomblin (Fomblin Profludropolyether, Ausimont, Thorofare, New Jersey, USA) for susceptibility matching and prevention of dehydration.

Images were acquired on a 7 Tesla MRI system (Bruker Biospin, Billerica, MA, USA) using a quadrature volume excitation coil (72 mm inner diameter) and a receive-only 4-channel phased array cryogenic coil. The specimens were imaged with the skull intact and placed in a syringe filled with Fomblin (perfluorinated polyether, Solvay Specialty Polymers USA, LLC, Alpharetta, GA, USA) to prevent tissue dehydration. 3D diffusion MRI data were acquired using a modified 3D diffusion-weighted gradient- and spin-echo (DW-GRASE) sequence (48) with the following parameters: echo time (TE)/repetition time (TR) = 30/400 ms; two signal averages; field of view (FOV) = 12.8 mm × 10 mm × 18 mm, resolution = 0.1 mm × 0.1 mm × 0.1 mm; two non-diffusion weighted images (b0s); 30 diffusion encoding directions; and *b* = 2,000 and 5,000 s/mm^2^, total 60 diffusion-weighted images (DWIs). Maps of fractional anisotropy (FA) were generated by tensor fitting using MRtrix (49). Co-registered T2-weighted (T2w) MRI data were acquired using a rapid acquisition with relaxation enhancement (RARE) sequence with the same FOV, resolution, and signal averages as the diffusion MRI acquisition and the following parameters: TE/TR = 50/3,000 ms, acceleration factor = 8; The total imaging time was approximately 12 h for each specimen.

### 2.3. Conventional CNN (cCNN), ResNet, and GAN based SR networks

**Figure 2:**
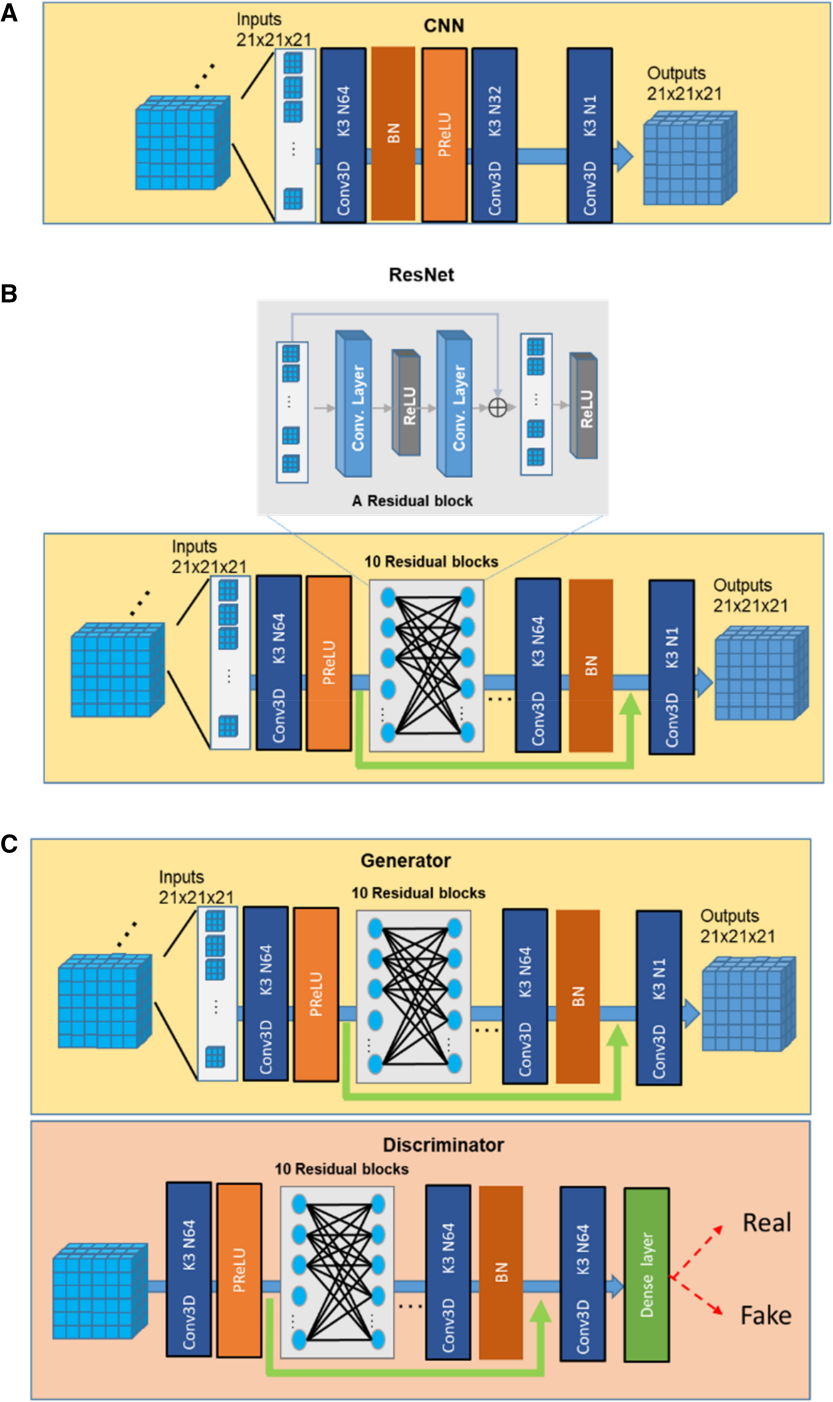
Basic network layouts of the cCNN (**A**), resNet (**B**), and GAN (**C**).

In our study, we compared the performance of three SR networks: cCNN, ResNet, and GAN. The basic architectures of the three types of networks are shown in Figure 2 and explained below.

#### 2.3.1. cCNN

The classical CNN models for SR used by previous studies (21) correspond to a specific parameterization of the baseline neural network architecture with 3 convolution layers, with two-dimensional (2D) kernel size 9, 1, 5 and channels 64, 32, 1, separately. In this study, we used the same architecture with the same number of channels but with 3D kernels with size of 3 for all layers (Figure 2A).

#### 2.3.2. Resnet

We followed the description in (23) to build a ResNet SR network. To achieve a balance between the performance and the cost of time and space, we used 10 Residual blocks and 64 channels for the convolution layer (50) and 3 × 3 × 3 filter size (Figure 2B). One residual block is composed of two convolutions, two ReLU with one additional layer.

#### 2.3.3. GAN

A typical GAN network consists of a generator and a discriminator (Figure 2C). In this study, we used the ResNet as the generator. The discriminator was composed of 10 Residual blocks and necessary dense, and Sigmoid layers (64 channels and 3 × 3 × 3 kernels in each layer). The discriminator was trained to identify the real high-resolution data from those generated from the Generator (51, 52).

One important consideration is the size of the input when training deep neural networks. Previous studies (e.g., 32), considered 2D and 3D training inputs separately. In our work, we used 2D data to test the effects of training samples size and 3D data to test the performance of SR as our MRI data were acquired in 3D. For training with 3D data, we chose 21 × 21 × 21 as the size of the input following the suggestion of (18). As for loss function, many previous works had used perception loss (5), which fits the human visual system. Nevertheless, considering the criteria of keeping the fidelity of image details, here we employed the simple mean square error (MSE) loss.

Due to the size difference between low and high-resolution images, up-sampling is necessary. With conventional camera picture super-resolution, many deep learning models incorporated transposed convolution for up-sampling under the assumption that natural scenery is locally continuous and smooth (10, 32, 53). However, considering the small input size used here, to minimize the edge effects, we up-sampled the 3D MRI volume before input to the neural network with cubic interpolation.

#### 2.3.4. Transfer learning

In order to fine-tune the network trained using mouse brain AF data and apply it on MRI data, we used transfer learning. This is necessary because high-resolution MRI data is scarce. We used transfer learning to obtain a specific network for the FA maps from diffusion MRI, by refining the ResNet network trained using AF data. By freezing most of the network layers' parameters while adjusting the last three layers weights by training with high-resolution FA maps from 6 mouse brains.

#### 2.3.5. Hyper-parameters for training

Other choices of hyper-parameters and tricks used during training procedures are primarily referring to practical considerations in (25). Rectified linear unit (ReLU) is used as the activation function that has the form *f*=max(0,*x*). The network is initialized with glorot_normal initialization (54) to encourage the weights to learn different input features. Glorot_normal initialization essentially draws form either a normal or uniform distribution, which is expressed in math formula as $W_{ij}∼U\left[-\frac{1}{\sqrt{n}},\frac{1}{\sqrt{n}}\right]$ (n is the number of columns in *W*, *U* is a uniform distribution), and keeps weights in a reasonable range across the entire network. The Adam method (55) is adopted for stochastic optimization in this study with beta1 of 0.9, beta2 of 0.999, epsilon of 1 × 10^−08^, and a learning rate of 0.001. The number of epochs is initialized as 1,000, but to prevent overfitting, early stopping is also employed when monitoring the validation set loss not decreasing in 100 epochs. The input data are preprocessed to standardize each input with the form of ranging [0, 1].

#### 2.3.6. Testing

We kept 10 from the 100 AF dataset for testing in AF SR experiments and 4 from 10 MRI data for testing in MRI SR experiments. All the testing data were not included in the training group. The testing was performed by dividing the brain into many 3D patches, with the size as the input of the deep neural network required, and recombine them together to reconstructive the entire 3D volumes. The quantitative evaluation was calculated based on selecting several group of brain slices with valid information from the reconstructed subjects and comparing with the corresponding high-resolution data.

#### 2.3.7. A ResNet network trained using human brain MRI data

We also conducted a study to evaluate whether a neural network trained using human brain MRI data can also be applied to mouse brain MRI data. We downloaded 60 3D humanbrain magnetization prepared rapid gradient echo (MPRAGE) MRI data from the HCP online database (https://humanconnectome.org/) (44) and down-sampled the data from the original 0.8 mm × 0.8 mm × 0.8 mm resolution to 1.6 mm × 1.6 mm × 1.6 mm resolution to train the ResNet network as described above. We tested the performance of the ResNet network on a separate group of down-sampled MPRAGE data (*n* = 5) and found the network can significantly improve the resolution of the images using the original data as the ground truth (Supplementary Figure S1). The SR performance of our network was comparable to previous reports.

### 2.4. Image quality and statistical analysis

We used root mean square error (RMSE) and structural similarity index (SSIM) to evaluate the difference between SR and reference images. RMSE was calculated by evaluating the absolute differences between two images (6), which rates pixel value fidelity, while SSIM appraises structure level fidelity (6).

We also used the open-access, parameter-free image resolution estimation tool developed by Descloux et al. (56) (https://github.com/Ades91/ImDecorr.git) to evaluate the effective image resolution. The algorithm utilizes image partial phase autocorrelation and uses the local maximum of the highest normalized frequency coefficient *K* (also termed cut-off frequency) to determine the effective resolution (effective resolution = voxel size/*K*).

Paired *t*-test in GraphPad Prism 9.0 (www.GraphPad.com) was used to test whether there was a significant difference between the RMSE/SSIM of images generated using different SR methods. Correlation analysis of AF and MRI signals was also performed using GraphPad Prism. Welch's ANOVA test in GraphPad was used to evaluate if the multiple population means are equal, and ultimately to assess whether the variable among different groups is a significant factor to the super-resolution.

## 3. Results

### 3.1. Comparisons of cCNN, ResNet, and GAN and required training dataset

**Figure 3:**
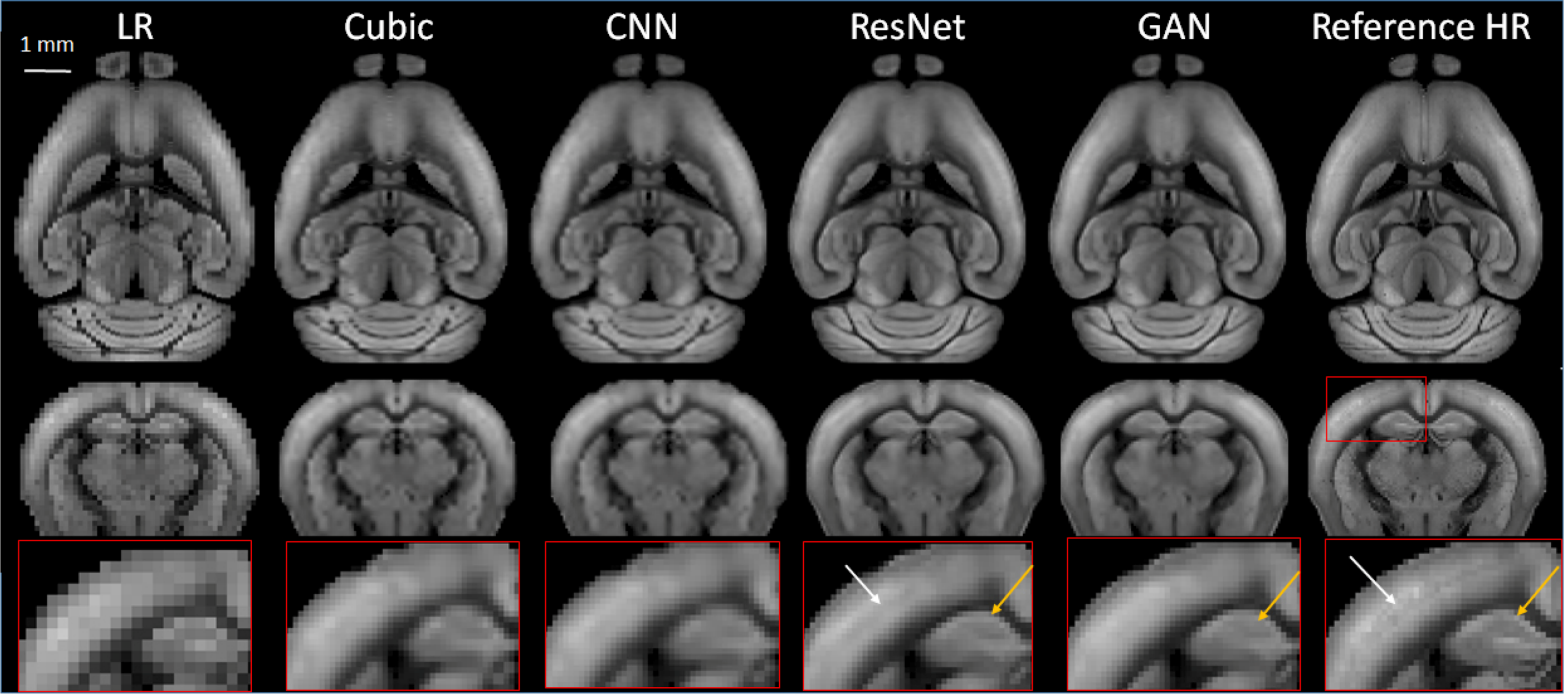
Representative SR images generated from down-sampled low-resolution images (LR, 200 µm isotropic) using cubic interpolation (cubic), cCNN, resNet, and GAN are compared with the reference image at 100 µm isotropic resolution. Top and middle panels: horizontal and axial images. Bottom panel: enlarged views of a region containing part of the cortex and hippocampus, as indicated by the red box in the axial reference image.

Using the large collection of 3D AF data from AMBCA, we first compared the performances of the three types of SR networks. We down-sampled the 3D mouse brain AF data from their native 25 µm resolution to 100 µm resolution and then to 200 µm resolution (Figure 1). We chose the 200 mm resolution here because *in vivo* MRI experiments often have spatial resolutions between 100 and 200 µm. After we completed training the networks using 20,000 21 × 21 × 21 patches of AF data (200 µm data as inputs and corresponding 100 µm data as training targets), a separate set of down-sampled 3D AF data (200 µm resolutions) (Figure 2, LR) were used for testing with the corresponding 100 µm resolution 3D AF data as the ground truth (Figure 3, Reference HR). Visually, the structural details in the SR results still did not match those in the reference 100 µm images. For example, the internal structures in the hippocampus were less clear in the SR results than in the reference (Figure 3, bottom row). The SR results generated by ResNet and GAN were closer to the reference 100 µm images than the results generated by cubic interpolation and cCNN.

**Figure 4:**
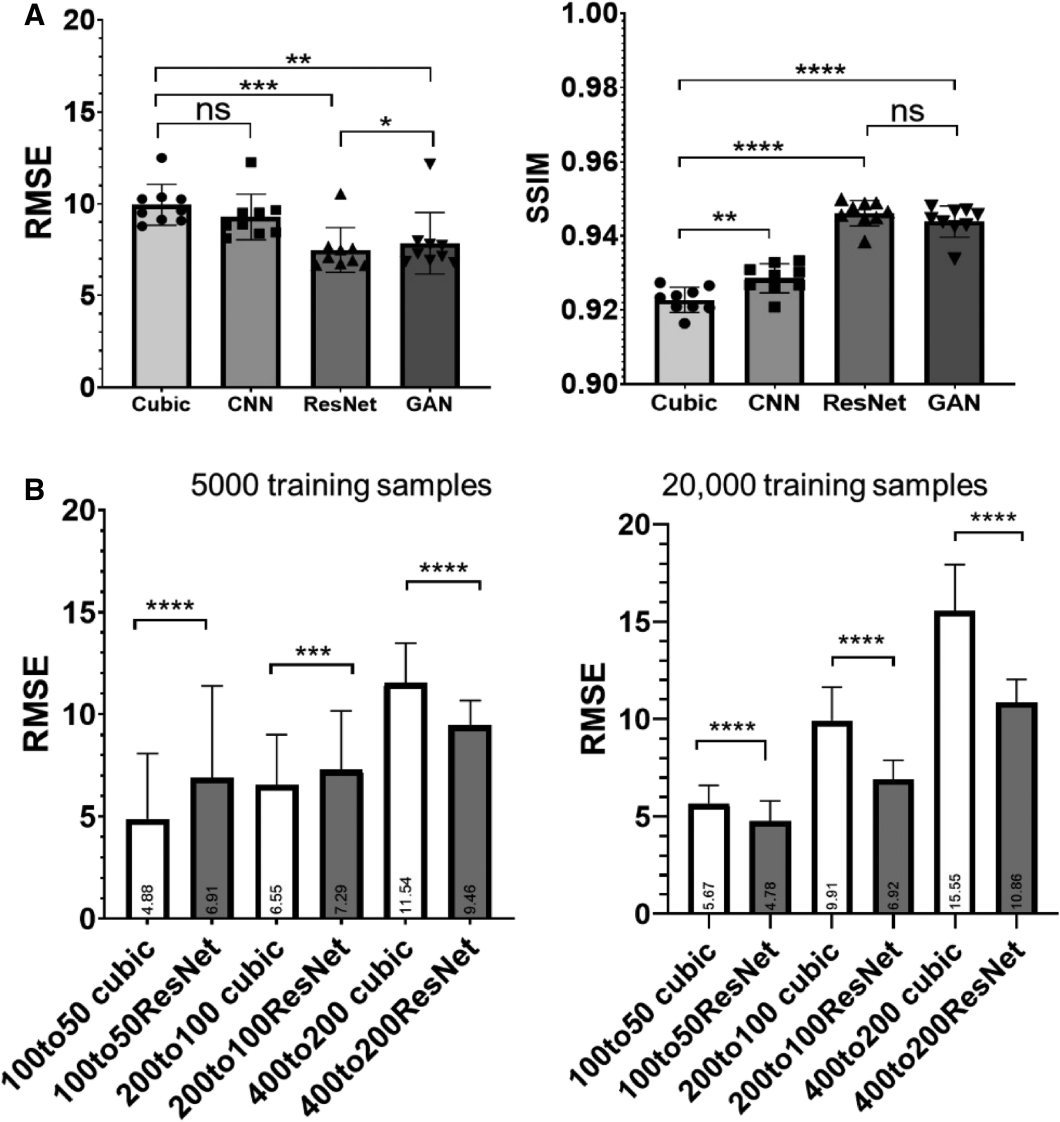
(**A**) Quantitative measurements (RMSE and SSIM) corresponding to Figure 3. (**B**) RMSE results by training and testing on various resolution/amount of auto fluorescence datasets. Left, results with 5,000 training samples. Right, results with 20,000 training samples.

RMSE analysis indicated ResNet produced the lowest RMSE, significantly outperforming cubic interpolation, GAN, and cCNN (Figure 4A). GAN yielded significantly lower RMSE values compared to cubic interpolation and cCNN. Regarding SSIM analysis, ResNet produced significantly higher SSIM values than cubic interpolation and cCNN, but no significant improvement over GAN (Figure 4A).

We also compared the ResNet SR results at different resolutions and training data size (listed in Table 1). With 5,000 training samples, the ResNet only out-performed cubic interpolation in terms of RMSE at the 400 µm resolution (*p* < 0.0001) and was out-performed by cubic interpolation at the 100 and 200 µm resolutions (Figure 4B). After increasing the number of training samples to 20,000, ResNet out-performed cubic interpolation at all three resolution levels (Figure 4B).

**Table 1:** Design and results of training on various resolution datasets.

Training data resolution (µm/voxel)	# of training samples	Improvement over cubic interpolation in RMSE
400–200	5,000	4.4 ± 1.1
200–100	5,000	−0.8 ± 0.9
100–50	5,000	−2 ± 1.5
400–200	20,000	4.7 ± 2.0
200–100	20,000	3.0 ± 1.4
100–50	20,000	2.1 ± 0.8

### 3.2. The role of training data resolution in SR

**Figure 5:**
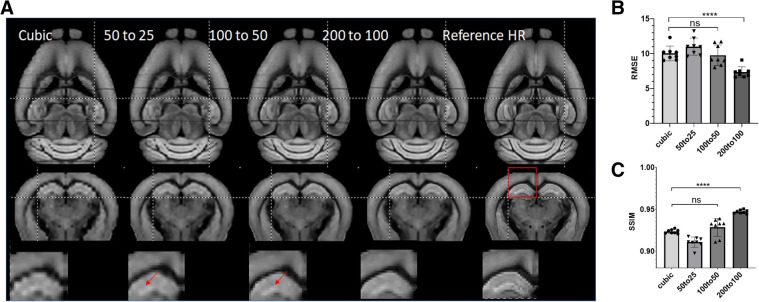
(**A**) Representative SR images generated from down-sampled low-resolution images (LR, 200 µm isotropic) using cubic interpolation (cubic) and resNet trained with images at three different resolutions and the reference image at 100 µm isotropic resolution. Top and middle panels: horizontal and axial images. Bottom panel: enlarged views of a region containing part of the cortex and hippocampus, as indicated by the red box in the axial reference image. (**B, C**) Quantitative measurements (RMSE and SSIM) of SR results in (**A**).

Next, we tested the generalization ability of SR networks trained at different resolutions. We trained three ResNet networks using data at 50–25, 100–50, and 200–100 µm, respectively, and tested their performances on 200 µm data. Visually, the network trained using 200–100 µm data performed better than networks trained using data at other resolutions (Figure 5A). Quantitative assessment showed that ResNet-based SR produced the best results when the resolution of the training data matched the resolution of actual data (Figures 5B,C). ResNet networks trained using higher resolution data did not produce significant improved RMSE and SSIM scores over cubic interpolation. The results generated by the ResNet network trained with matching resolution (200–100 µm) improved RMSE and SSIM significantly (ANOVA, *p* = 0.0019, *F* = 7), highlighting the importance of resolution for training data.

### 3.3. Cross-modality transfer learning SR

**Figure 6:**
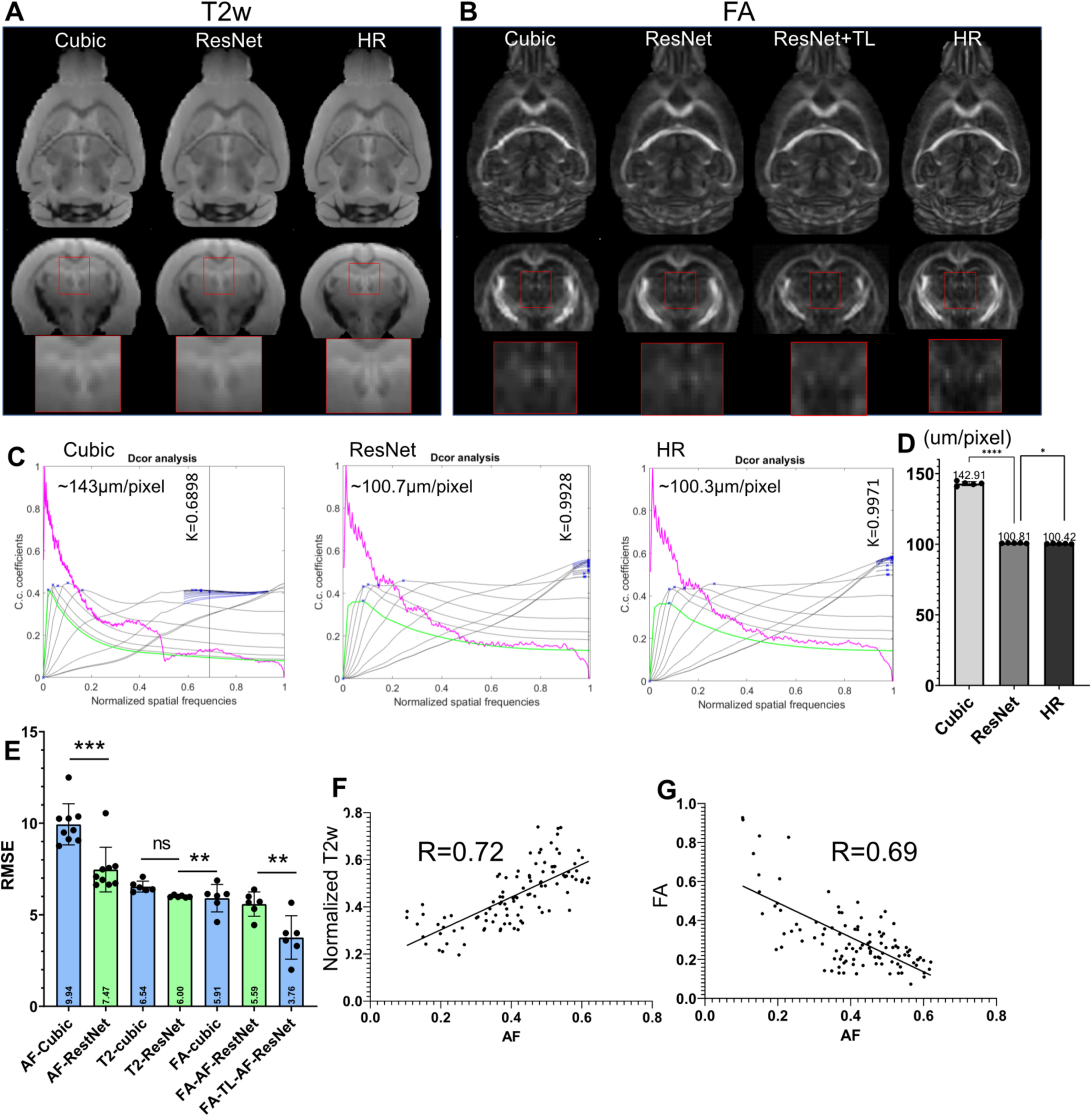
Super-resolution of T2-weighted (T2w) and FA images of the mouse brain. (**A**) T2w MRI SR using the ResNet trained using the 3D AF data compared with cubic interpolation results and high-resolution (HR) data; (**B**) results of FA SR using the ResNet trained on the AF data (ResNet) and transfer learning (ResNet + TL) compared with cubic interpolation results and HR data. (**C**) Estimation of the effective resolutions of the cubic interpolation, ResNet SR, and HR T2w images shown in (**A**). The magenta lines: radial average of log of the absolute value of Fourier transform from T2w images; the gray lines: all high-pass filtered decorrelation functions; blue to black lines: decorrelation functions with refined mask radius and high-pass filtering range. Blue crosses: all local maxima. *K* is the max of all maxima (shown as a vertical line) and the cut-off frequency. The effective resolution was calculated as nominal resolution (100 µm here) divided by *K*. (**D**) Effective resolution of the cubic-interpolation, ResNet SR, and native HR images (*n* = 5). (**E**) RMSE results of selected images shown in (**A,B**). (**F,G**) Voxel-wise correlations between AF and normalized T2w as well as between AF and FA signals.

Although the previous results showed effective SR using the 3D AF data from AMBCA, using the same approach to SR mouse brain MRI data remained challenging due to the lack of a large high-resolution mouse brain MRI dataset. One question is whether we can use the network trained on the 3D AF data to SR mouse brain MRI data. To test this approach, we down-sampled 3D T2-weighted (T2w) and FA MR images of the mouse brain from 100 µm resolution to 200 µm resolution and applied the ResNet network trained using 200–100 µm 3D AF data. The results were compared to the original 100 µm FA and T2w MR images. Visually, the ResNet results were better than cubic interpolation results for both FA and T2w (Figures 6A,B), comparing the jagged edges in cubic interpolated results with smooth edges in ResNet results. Quantitative assessment showed that the ResNet T2w MRI results indeed had higher effective resolution, measured using a toolbox in (56), and lower RMSE than the cubic interpolated T2w MRI data (Figures 6C–E). However, the RMSE of the SR FA data showed no improvement over the cubic interpolation results (Figure 6E). The difference between T2w and FA images might be explained by the difference in MR contrasts. After co-register the FA and T2w data with the 3D AF data, the AF signals showed a positive correlation with normalized T2w (*R* = 0.72, *p* < 0.0001) and a negative correlation with FA values (*R* = 0.69, *p* < 0.0001), as shown in Figures 6F,G.

We then used transfer learning by keeping on training the last three layers of the neural network while fixing the rest of the network. Compared with results from directly applying the network trained using AF data, the SR result from transfer learning showed significantly reduced RMSE (Figure 6E). This result suggests that transfer learning may allow us to use SR network trained using 3D AF data to SR MRI data.

### 3.4. Training from human MRI and application on mouse MRI

**Figure 7:**
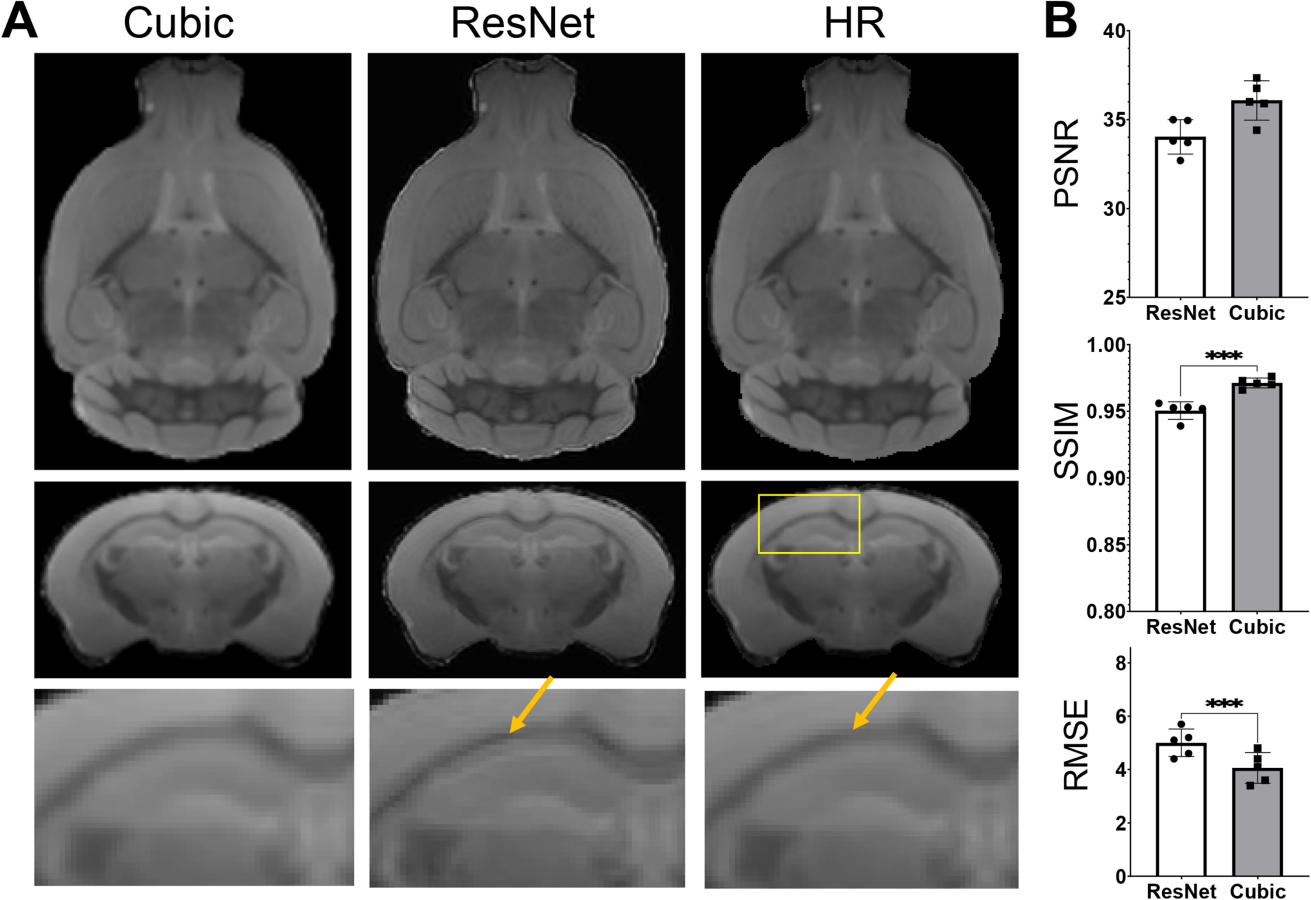
Super-resolution of T2w images of the mouse brain using the resNet trained using HCP MPRAGE data. (**A**) T2w MRI SR using the ResNet trained using the 3D AF data compared with cubic interpolation results and high-resolution (HR) data; (**B**) the PSNR, SSIM, and RMSE of the results from cubic-interpolation and ResNet.

As there have been many reports on human brain MRI SR (26, 57, 58), it is worth accessing whether the SR networks trained using human brain MRI data can be applied to mouse brain MRI data. Apply the ResNet network trained using HCP MPRAGE data to the mouse brain T2w MRI data generated sharper results than cubic interpolation (Figure 7), but also introduced some ringing artifacts (indicated by the orange arrows in Figure 7A). Quantitative evaluations using PSNR, SSIM, and RMSE suggested that the human ResNet results were significantly worse than cubic interpolation (Figure 7B).

## 4. Discussion

The goal of our study is to investigate whether deep learning-based SR can enhance the resolution of mouse brain MRI data. Acquiring high-resolution MRI data of the mouse brain or human brain is technically challenging and requires lengthy acquisition. For example, Wang et al. recently demonstrated dMRI of the mouse brain at 25 µm isotropic resolution, which took 95 h even with compressed sensing (59). Prolonged MRI acquisition is expensive and impractical due to the constant drift of the magnetic field as well as sample stability. Moreover, the available resolution also depends on the MRI contrast. For example, certain MRI acquisition techniques (e.g., dMRI) inherently require lengthy acquisition and have lower signal-to-noise (SNR) than conventional T1- and T2-weighted MRI. As a result, MRI data acquired using such techniques often have limited spatial resolutions and could benefit from SR methods.

In this study, we compared three types of deep learning networks for SR. As expected, ResNet and GAN outperformed cCNN in this study. This is because cCNNs require a large number of parameters to accommodate all potential features in images, which can lead to overfitting, as reported in previous studies (52, 60). The finding that ResNet outperformed GAN, however, was surprising to us because GAN has shown improved performances in many conventional image processing tasks including realistic image generation (61), inpainting (62), and image repairing (63). One possible explanation is that the loss functions used by the generator and discriminator here were not designed specifically to minimize the RMSE. In a previous study (52), a GAN network similar to the one used in this study generated visually realistic results but had lower RMSE and SSIM values against the ground truth than ResNet. Recent developments in GAN networks, such as conditional GAN (64), Cycle GAN (65), and the use of more sophisticated loss functions instead of the simple MSE loss function, may improve the performance of GAN for SR in the future.

Previous work on SR of generic images did not consider the effects of actual image resolution as the training and testing images were often acquired under a wide range of settings (e.g., multiple subjects in a scene and varying image sizes). However, for MRI and medical imaging, imaging resolution can be precisely defined by the field of view and image dimension and is an important parameter for evaluating its diagnostic value. Our results based on the mouse brain AF data demonstrate that the resolutions of the training data need to match the target resolution in order to achieve optimal SR performance and mismatches between the resolutions of training and target data resulted in reduced SR performance. In MRI data, structural details visible at different resolutions may have distinct statistical characteristics. For example, at low resolution (200–400 µm), we can only see major white matter tracts in the mouse brain, but as the resolution improves, smaller white matter tracts and other structural features emerge. Although it is possible to train a network with data of multiple resolutions, doing so will result in increased network complexity and require more training data.

We showed that increasing the training data size from 5,000 to 20,000 improved the network performance, but there is no hard threshold as the amount of training data needed depends on the network as well as the data pattern. For generic image SR, an early report on CNN-based SR used only 91 images, divided into 24,800 training samples, and the authors claimed that the training set already captured enough nature images features to avoid overfitting (23). Another study (43) used 5,744 slices to train a GAN-based SR network. In our case, the ResNet had about more than 10,000 parameters, and the 20,000 training samples were likely to capture all the mouse brain features to prevent overfitting. However, given the large number of optical data in AMBCA, we can potentially further increase the number of the training dataset.

As there is a lack of large collections (100 or more subjects) of high-resolution mouse brain MRI data, we investigated whether the extensive set of AMBCA mouse brain optical data could be used for SR of mouse brain MRI data. However, we found that the direct application of the SR network trained using AF to MRI data did not work well and transfer learning was required. Our results indicate that direct training on AF data can enhance the T2w MRI data, but provides limited improvement in the resolution of FA images. This may be due to the anisotropic microstructure organization characterized by FA, which may not be captured by the AF images. Transfer learning may have worked in this case because FA and AF images share links to some common features. It has also been reported that the contrast in AF mainly reflects tissue myelin content, and as myelinated white matter structures have anisotropic microstructure, there is a potential indirect link between AF and FA contrasts. However, it is important to note that high-resolution MRI data still needs to be collected to train the network.

One critical question of deep learning-based SR is whether it can be extended to human brain MRI data. Several studies have used SR techniques to improve the resolution of human brain images. For example, in (66), SR was used to enhance the low-resolution spectroscopic images of the human brain to detect the metabolic features. Other studies, such as (67), have proposed to apply SR models trained using lower resolution data (e.g., 2.5–1.25 mm) to high resolution data (1.25 mm or higher). However, our results in Sections 3.2 and 3.4 demonstrate that the SR becomes ineffective once the resolution and contrast discrepancy between the training and target data increases beyond a certain extent. For the current deep learning SR methods, the lack of high-resolution training data remains a significant challenge, which may be alleviated by using images acquired from postmortem brain specimens, taking into consideration the differences in MRI signals between *in vivo* and post-mortem MRI. Future studies are needed to explore the generalizability of deep learning-based SR methods to human brain MRI data with different image contrasts and imaging protocols, as well as the potential of using transfer learning and data augmentation techniques to enhance the performance of SR models.

In summary, we demonstrated that a deep learning network trained using AF data acquired from the mouse brain using serial two photon microscopy can improve the resolution of mouse brain MRI data via transfer learning. Our results suggest that the deep learning network can achieve better MRI SR than conventional cubic interpolation. This approach potentially allows us to leverage the large collection of mouse brain data from AMBCA and reduce the requirement on available high-resolution MRI data.

## Data Availability

The raw data supporting the conclusions of this article will be made available by the authors, without undue reservation.
