# Supplementary material for: Mouse brain MR super-resolution using a deep learning network trained with optical imaging data
Source: Front Radiol. 2023 May 15;3:1155866. doi: 10.3389/fradi.2023.1155866 (PMC10365285; doi:10.3389/fradi.2023.1155866)
Supplement: Supplementary file 1 [file Datasheet1.docx]

Supplementary Material

Mouse Brain MR Super-Resolution Using a Deep Learning Network Trained with Optical Imaging Data

Zifei Liang^1^, Jiangyang Zhang^1*^

*** Correspondence:** Corresponding Author: Jiangyang.Zhang@nyulangone.org

#

**Fig.S1:** The performance of the ResNet trained using HCP MPRAGE data. **A**. Comparisons between cubic-interpolation, ResNet, and high-resolution (HR) data showing the coronal (top) and axial (middle) planes as well as zoom-in views of the occipital area (bottom); **B.** PSNR, SSIM, and RMSE results of images shown in A.***: p<0.001 from paired *t*-test; ****: p<0.0001 from paired *t*-test

**
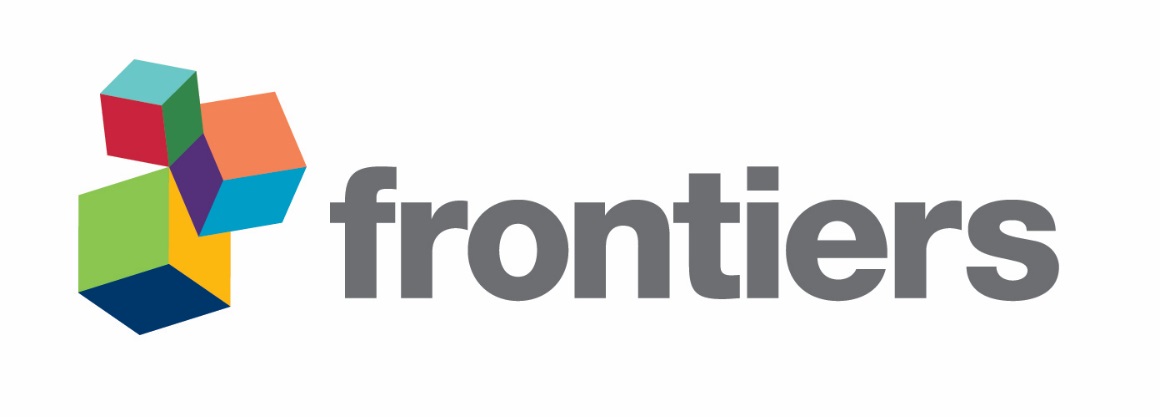
**
